# Identification of Hub Genes and Small Molecule Drugs Associated with Acquired Resistance to Gefitinib in Non-Small Cell Lung Cancer

**DOI:** 10.7150/jca.56506

**Published:** 2021-07-02

**Authors:** Guangda Li, Yunfei Ma, Mingwei Yu, Xiaoxiao Li, Xinjie Chen, Yu Gao, Peiyu Cheng, Ganlin Zhang, Xiaomin Wang

**Affiliations:** 1 Beijing Hospital of Traditional Chinese Medicine, Capital Medical University, Beijing, China.; 2 Beijing University of Chinese Medicine, Beijing, China.

**Keywords:** bioinformatics analysis, gefitinib resistance, non-small cell lung cancer, WGCNA, hub genes, GSEA.

## Abstract

Targeting EGFR, epidermal growth factor receptor tyrosine kinase inhibitors (EGFR TKIs), brings lights to the treatment of non-small cell lung cancer (NSCLC). Although T790M mutation responded as one of the main reasons of acquired resistance, still 15% of the resistance patients can't be explained by the known mechanisms. The purpose of this research was to identify some new mechanisms of gefitinib acquired resistance, and to predict small molecules drugs which may reverse drug resistance by integrated bioinformatics analysis. The GSE34228 data package containing the microarray data of acquired gefitinib-resistant cell line (PC9GR) and gefitinib-sensitive cell line (PC9) from the GEO database were downloaded, and gene co-expression networks by weighted gene co-expression network analysis (WGCNA) were constructed to identified key modules and key genes related to gefitinib resistance. Furthermore, the significantly differentially expressed genes (DEGs) between the two cell types were screened out, and a protein-protein interaction (PPI) network to obtain the key genes of DEGs was accordingly constructed. Through the above two methods, 4 hub genes, PI3, S100A8, AXL and PNPLA4 were mined as the most relevant to gefitinib resistance. Among them, PI3, S100A8 were down-regulated in PC9GR cell samples, while AXL, PNPLA4 were up-regulated. The gene set enrichment analysis (GSEA) for single gene showed that the four hub genes were mainly correlated with cell proliferation and cycle. Besides, small molecule drugs with the potential to overcome resistance, such as Emetine and cephaeline, were screened by CMap database. Consistent with this, *in vitro* experiments results have shown that emetine and cephaeline can increase the sensitivity of drug-resistant cells to gefitinib, and the mechanism may be related to the regulation of PI3 and S100A8. In conclusion, 4 hub genes were found to be related to the occurrence of gefitinib resistance in non-small cell lung cancer, and several small molecule drugs were screened out as potential therapeutic agents to overcome gefitinib resistance, which may lead a new way for the treatment of NSCLC of acquired resistance to gefitinib.

## Introduction

Lung cancer ranks first among the cause of cancer-related mortality 1, while non-small cell lung cancer (NSCLC) accounts for approximately 85% of lung cancers 2. In recent years, the discovery of gene mutations has developed individualized targeted therapy, especially for NSCLC patients with epidermal growth factor receptor (EGFR) mutation-positive, epidermal growth factor receptor tyrosine kinase inhibitors (EGFR TKIs) provide a good treatment outcome, but the problem of resistance has not been fully solved. Gefitinib, the first-generation EGFR TKI, is one of the most common treatments for NSCLC with EGFR mutations. But almost all patients will develop acquired resistance within about a year 3, 4. 50% of patients with acquired resistance has been discovered having T790M mutation 5, 6, some others are reported with activation of alternative pathways or downstream pathways, histological or phenotypic transformation 7, but still 15% patients with acquired resistance cannot be attributed to the above mechanism 8. With the third generation EGFR TKI, such as Osimertinib, which having been developed to overcome EGFR T790M, half patients with acquired resistance could benefit, however, the acquired resistance will still inevitably occurs after treatment with Osimertinib. At the same time, for the follow-up treatment of patients with other acquired resistance mechanisms, chemotherapy, combination therapy, or immunotherapy may be considered, but optimal treatment is not yet clearly defined 8. It is necessary to find some new molecular mechanisms of gefitinib acquired resistance and new small molecule drugs that may reverse resistance.

At present, the development of microarray and sequencing technology has provided help to further explore the molecular mechanism of tumors, and a variety of bioinformatics approaches have also been widely used. Weighted gene co-expression network analysis (WGCNA) is based on systems biology approach to determine correlations among genes 9, and co-expression modules and key genes that are highly correlated with phenotypic traits are obtained. Currently, WGCNA has been used in many studies to find therapeutic targets and candidate biomarkers for various tumors 10-12.

In the present study, hub genes related to gefitinib acquired resistance in NSCLC were identified by combining WGCNA and differential gene expression analysis, and functional enrichment analysis was performed for key modules in co-expression network. Meanwhile, the CMap database and *in vitro* experiments was used to predict and verify small molecule drugs that may overcome the acquired resistance to gefitinib in NSCLC.

## Materials and methods

### Data collection and preprocessing

**Figure 1:**
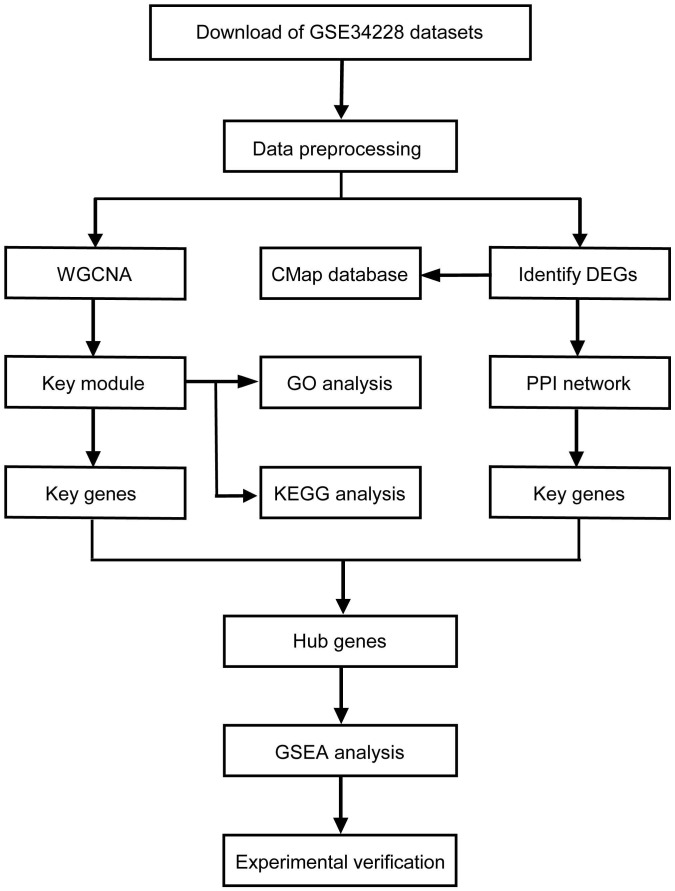
** Study workflow.** WGCNA, weighted gene co-expression network analysis; GO, Gene Ontology; KEGG, Kyoto Encyclopedia of Genes and Genomes; DEG, differentially expressed genes; PPI, protein-protein Interaction; GSEA, gene set enrichment analysis.

The mRNA expression profiles of human non-small cell lung cancer with acquired gefitinib-resistant were downloaded from the Gene Expression Omnibus (GEO) database. GSE34228 was based on Agilent-014850 Whole Human Genome Microarray and included 208 samples, which were treated with the four different conditions: EGF-treatment, gefitinib-treatment, both EGF and gefitinib-treatment and no treatment 13. We then picked out 52 untreated samples, including 26 PC9GR (acquired gefitinib-resistant) cell samples and 26 PC9 (gefitinib-sensitive) cell samples for further analysis. The normalized data was downloaded and the matrix of gene expression was obtained. Then mapped all gene probes to gene symbols by using the microarray annotations, the average expression value was calculated out for those genes with corresponding to multiple probes, and the probe without corresponding annotation information were removed. Finally, 19,749 genes were retained from the 45,220 genes in the dataset for subsequent analysis. The flowchart of this study was showed in **Figure 1**.

### Construction of co-expression network and identification of significant modules

A total of 4937 genes in the top 25% of variance were selected from 19749 genes to construct co-expression networks, and the R package “WGCNA” was applied to screen out the modules most related to gefitinib resistance and the hub genes among them 14. We first set soft-thresholding power as 7 when 0.8 was used as the correlation coefficient threshold, and transform the adjacency matrix into a topological overlap matrix (TOM) 15. Then, according to the TOM-based dissimilarity measurement, hierarchical clustering was conducted to classify similar genes into gene modules with a minimum size of 30 for the gene dendrogram. In order to merge highly similar modules, we calculated module eigengenes and defined 0.25 as the threshold for cut height. The key module was defined as the module most relevant to gefitinib resistance, and the key genes in the module was screened out with gene significance (GS) and module membership (MM) both greater than 0.9.

### Function enrichment analyses

To further understand the function of genes in the module most related to gefitinib resistance, Gene Ontology (GO) enrichment and Kyoto Encyclopedia of Genes and Genomes (KEGG) pathway was analyzed using the R package “clusterprofiler” 16, and p-value <0.05 was considered to be significant enrichment.

### DEGs identification

The R package “limma” was performed for DEGs identifying between PC9GR cell samples resistant to gefitinib and PC9 cell samples sensitive to gefitinib 17, 18, and the significantly altered genes was selected with p-value <0.05 and |log2 fold change (FC)| ≥2.

### PPI network construction

We uploaded the selected DEGs to the Search Tool for the Retrieval of Interacting Genes (STRING) database to build a PPI network 19, and the medium confidence score >0.4 was considered significant. Cytoscape software was used to visualize the PPI network, and genes with connectivity degree ≥5 were defined as key genes.

### Hub gene identification and GSEA

Key genes that belong to both the co-expression network and the PPI network were considered as hub gene for further analysis. To explore the role of the hub genes in gefitinib resistance, the R package “clusterprofiler” was used for gene set enrichment analysis of single hub gene 16. According to the median expression of each hub gene, 26 PC9GR cell samples were divided into high expression group and low expression group, annotated gene set “c2.cp.kegg.v7.1.symbols.gmt” was selected as the reference gene set downloaded from the Molecular Signatures Database (MSigDB) 20, and p-value <0.05 was considered significant. Then we used the R package “enrichplot” to visualize the top 3 gene sets in the enrichment score.

### Identification of candidate small molecules

To identify potential small molecule drugs that may reverse gefitinib resistance, we uploaded the top 150 up-regulated and down-regulated DEGs into CMap clue.io database (L1000 platform) 21, and compared our gefitinib resistance signature with the expression profiles from 9 human cancer cell lines treated with reference drugs. Finally, a list of drugs was generated, high negative median score indicates that the drug reversed the expression of our query signature.

### Cell viability assay

The MTT assay was used to assess cell viability. After the cells were treated with different concentrations of drugs in 96-well plates for 48 hours, 20 µL MTT reagent (Sigma-Aldrich, USA) were added to each well and cells were cultured at 37 ℃ incubator for 4 hours. Then, the original media was removed, and 150 µL of DMSO was added to each well and shaken for 1 min, OD value was measured at 570 nm with a microplate reader (Thermo Fisher Scientific, USA).

### RNA Extraction and quantitative real-time PCR (qRT-PCR)

Total RNA from cells was extraction using the total RNA extraction kit (spin column, TIANGEN, China) following the manufacturers' instructions. Then we carried out complementary DNA (cDNA) synthesis with the first-strand cDNA kit (TIANGEN, China). Quantitative real-time PCR (qRT-PCR) analysis was performed in the CFX Connect Real-time System (Bio-Rad, USA) using the SYBR Green qPCR Master Mix (Bimake, USA). PCR conditions were: 95 °C for 15 min, 40 cycles at 95 °C for 10s, 55 °C for 30s and extension at 72 °C for 30s. Relative mRNA expression was calculated using the 2^-ΔΔ*Ct*^ method and normalized to glyceraldehyde 3-phosphate dehydrogenase (GAPDH). All sequences of the primers are as follows: PI3 (forward primer: 5'-AGATCCCGTTAAAGGACAAGTT-3', reverse primer: 5'-GTATCTTTCAAGCAGCGGTTAG-3'); S100A8 (forward primer: 5'-TATCATCGACGTCTACCACAAG-3', reverse primer: 5'-TCTGCACCCTTTTTCCTGATAT-3').

## Results

### Identification of key modules and key genes by WGCNA

**Figure 2:**
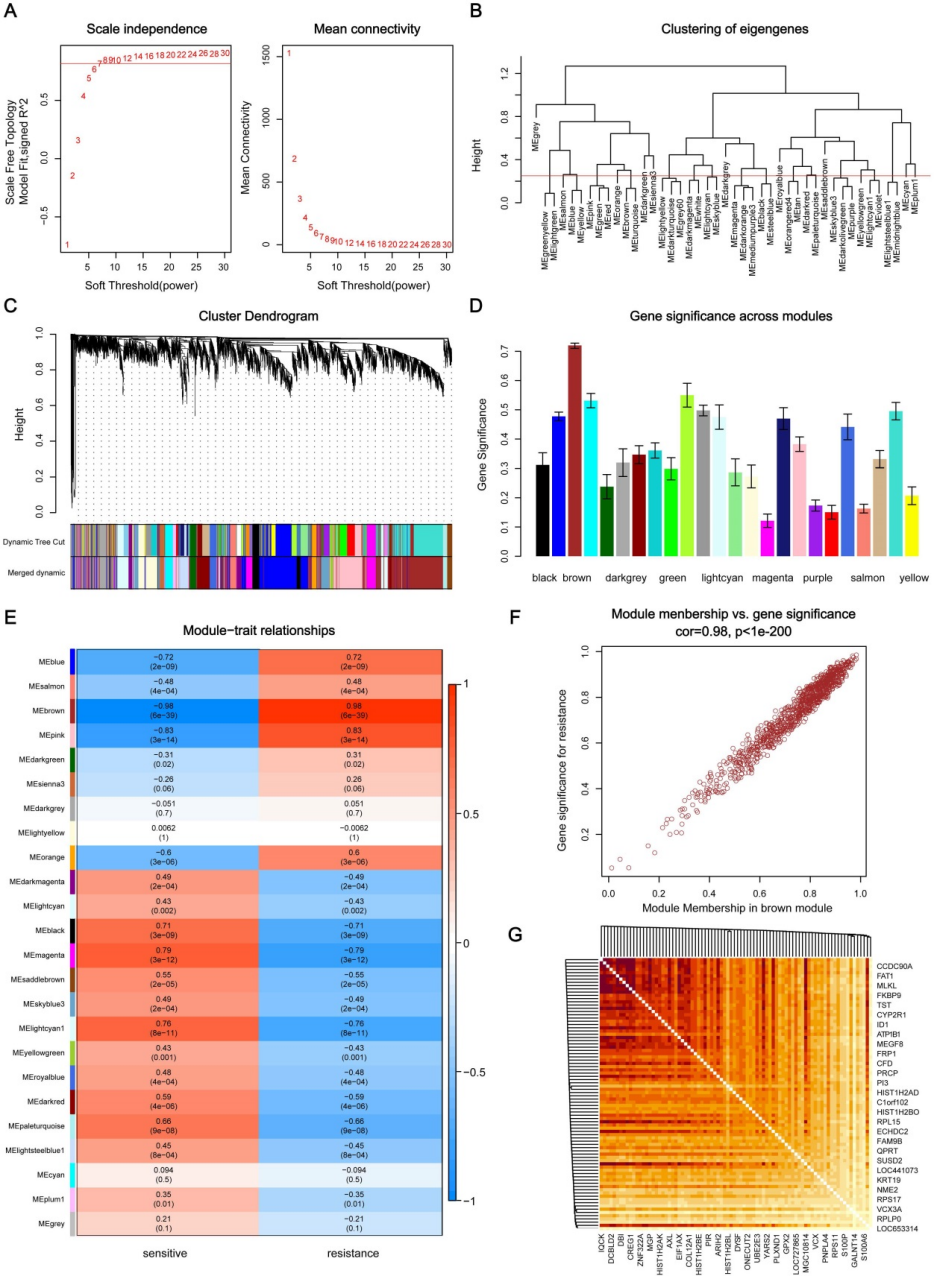
** Identification of key modules and key genes associated with acquired resistance to gefitinib through WGCNA.** (A) Analysis of the scale-free fit index and the mean connectivity for various soft-thresholding powers. (B) Cluster dendrogram based on module eigengenes (cutting height = 0.25). (C) Dendrogram of all DEGs in the top 25% of variance clustered based on the measurement of dissimilarity (1-TOM). The color band indicates the initial modules and the merged modules. (D) Distribution of average gene significance and errors in the modules associated with gefitinib resistance. (E) Heatmap of the correlation between module eigengenes and gefitinib resistance. (F) Scatter plot of eigengenes in brown module. (G) 84 key genes in brown module were tightly associated with each other. WGCNA, weighted gene co-expression network analysis; DEG, differentially expressed genes; TOM, topological overlap matrix.

Through the analysis of scale independence and mean connectivity, soft-thresholding power of 7 (scale free R^2^=0.8) was selected to ensure a scale-free network (**Figure 2A**), and the modules with a correlation higher than 0.75 were merged, ultimately, a total of 24 co-expression modules were recognized (**Figure 2B-C**), ranged in size from 55 to 1016 genes. The average gene significance in each module (**Figure 2D**) and the correlation between module eigengenes and gefitinib resistance (**Figure 2E**) both indicated that brown module was the key module highly related to gefitinib resistance. The brown module contained a total of 1016 genes, 84 genes were selected under the condition that both GS and MM were greater than 0.9 (**Figure 2F**), and these genes were also closely related to each other (**Figure 2G**). Thus, these genes were considered as key genes for further validation analysis.

### Functional annotation of the key modules

**Figure 3:**
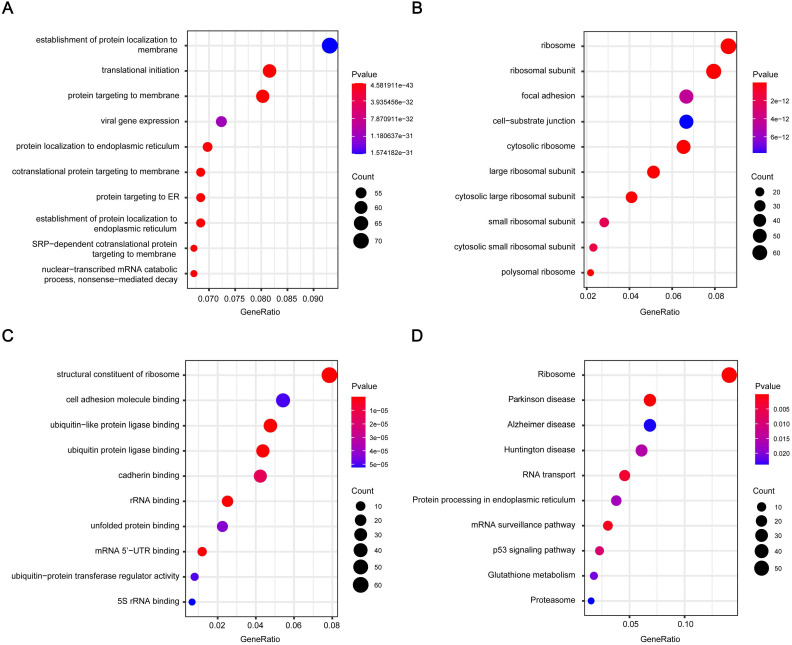
** GO and pathway enrichment analysis of the brown module genes.** (A) Biological process analysis. (B) Cellular component analysis. (C) Molecular function analysis. (D) KEGG pathway analysis. GO, Gene Ontology; KEGG, Kyoto Encyclopedia of Genes and Genomes.

1016 genes in brown module were subjected to GO functional and KEGG pathway enrichment analyses, and the result was showed in **Figure 3**. GO analysis results showed genes in the brown module were mainly associated with the biological processes of protein generation and transport, such as establishment of protein localization to endoplasmic reticulum, translational initiation, and protein targeting to membrane (**Figure 3A**). In KEGG pathway analysis, protein production and transport pathways are also enriched, including Ribosome, RNA transport, and Protein processing in endoplasmic reticulum. What's more, some pathways related to Neurodegenerative disorders of ageing were enriched (**Figure 3D**).

### Identification of DEGs and PPI network construction

**Figure 4:**
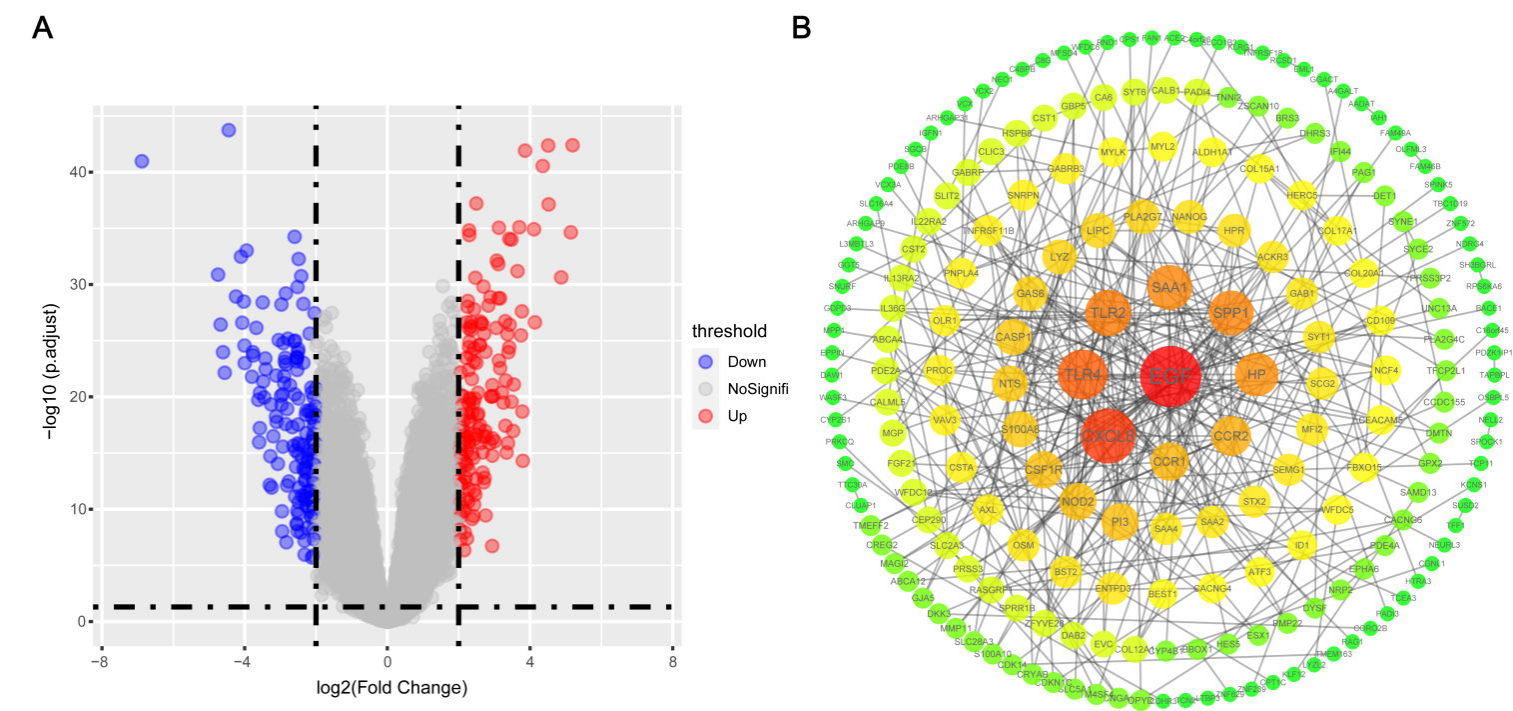
**DEGs and PPI network construction.** (A) Volcano map of DEGs between gefitinib-resistant PC9GR cells and gefitinib-sensitive PC9 cells. (B) PPI network analysis of filtered DEGs. PPI, protein-protein interaction.

A total of 320 DEGs were screened, including 160 up-regulated genes and 160 down-regulated genes in gefitinib-resistance PC9GR cells compared with gefitinib-sensitive PC9 cells (**Figure 4A**). Then, we constructed a PPI network with 207 nodes and 347 edges for all 320 DEGs by Cytoscape according to the STRING database (**Figure 4B**), and 42 genes with degree ≥5 were identified as key genes.

### Identification of hub genes and GESA

**Figure 5:**
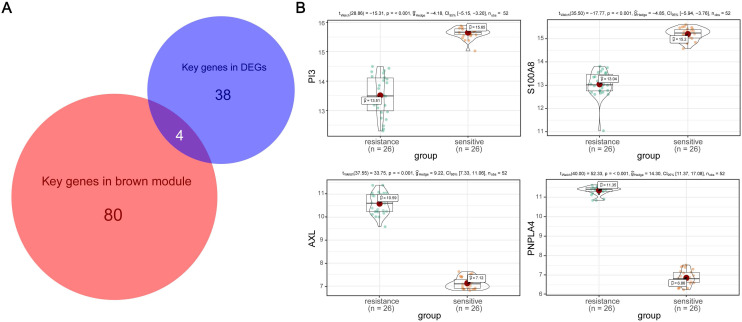
** Hub gene detection.** (A) Identification of common key genes between DEGs and the brown module by overlapping them. (B) PI3, S100A8, AXL, and PNPLA4 gene expression differences between gefitinib-resistant PC9GR cells and gefitinib-sensitive PC9 cells.

According to the key genes in brown module and the key genes in DEGs, 4 overlapping genes were identified as hub genes (**Figure 5A**), which are PI3, S100A8, AXL and PNPLA4. The expression of PI3 and S100A8 were significantly down-regulated in PC9GR cell samples compared to PC9 cell samples, conversely, the expression of AXL and PNPLA4 were significantly up-regulated (**Figure 5B**).

**Figure 6:**
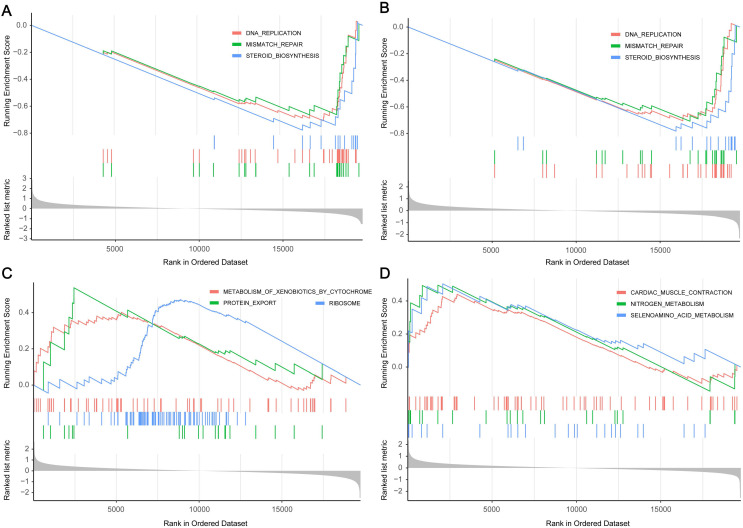
** Gene set enrichment analysis of hub genes. (A)** The top three enriched pathways in PI3 low-expression group. **(B)** The top three enriched pathways in S100A8 low-expression group. **(C)** The top three enriched pathways in AXL high-expression group. **(D)** The top three enriched pathways in PNPLA high-expression group.

Next, through GSEA of the four hub genes, we found that genes in low expression groups of PI3, S100A8 and high expression groups of AXL, PNPLA4 were all enriched in genetic information processing and metabolism pathways. Gene set with higher enrichment scores included DNA replication, Mismatch repair, Protein export, Steroid biosynthesis, Selenoamino acid metabolism (**Figure 6**).

### Related small molecule drugs screening

After importing the DEGs into the Connectivity Map (L1000 platform), a drug list was generated. **Table 1** shows 10 small molecule drugs with the median score <-60 that may reverse the resistance of gefitinib. The top negative enrichment scores belong to Emetine, PU-H71, and cephaeline. In these drugs, protein synthesis inhibitors play an important role.

### *In vitro* validation of small molecule drugs

**Figure 7:**
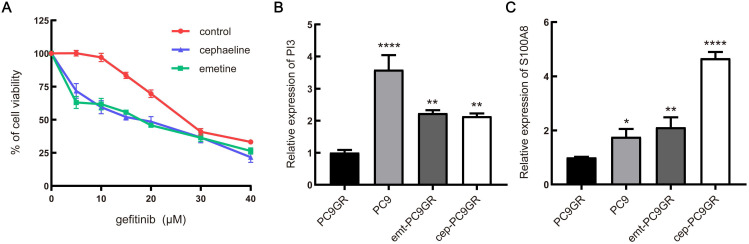
***In vitro* validation of small molecule drugs. (A)** MTT cell viability analysis of PC9GR cells treated with combination of emetine (3 nM) or cephaeline (1 nM) and various concentration gefitinib for 48 h. **(B-C)** mRNA expression of PI3 and S100A8 in different groups of cells. emt-PC9GR: PC9GR cells treated with emetine (67 nM); cep-PC9GR: PC9GR cells treated with cephaeline (13 nM); * indicated P < 0.05, ** indicated P < 0.01, **** indicated P < 0.0001 compared to PC9GR cell group.

To determine the correlation between the predicted drug and gefitinib resistance, we screened out two protein inhibitors with a score higher than 70 for verification. First, PC9GR cells were treated with combination of emetine (3 nM, the IC_10_ value of emetine in PC9GR cells) or cephaeline (1 nM, the IC_10_ value of cephaeline in PC9GR cells) and various concentration of gefitinib for 48h. Our results demonstrated that PC9GR cells interfered with emetine and cephaeline were more sensitive to gefitinib, the IC_50_ value decreased from (28.41 ± 0.17) μM to (14.73 ± 1.64) μM、(15.13 ± 1.58) μM (**Figure 7A**).

Next, we examined the underlying mechanism of the above phenotypes by qRT-PCR. It was found that two down-regulated genes of the four predicted genes showed significant changes with the intervention of emetine and cephaeline. The mRNA expression of PI3 and S100A8 genes in sensitive cells was significantly higher than that in resistant cells, this is consistent with our previous prediction. At the same time, emetine (67 nM) and cephaeline (13 nM) significantly increased the mRNA expression of PI3 and S100A8 in PC9GR cells (**Figure 7B-C**).

## Discussion

Acquired resistance to gefitinib is a complex biological process. In this study, using publicly available microarray data, four hub genes related to gefitinib resistance were identified through a series of bioinformatics methods. Through GSEA, the potential functions of 4 hub genes in gefitinib resistance were explored, and several potential small molecule drugs that can reverse gefitinib resistance were screened.

The 4 hub genes consist of PI3, S100A8, AXL and PNPLA4. Except for AXL, the other three genes have not been reported related to gefitinib resistance. PI3, encoding elafin, an endogenous serine protease inhibitor 22. Elafin is a member of the WAP four-disulfide-core (WFDC) family 23, which has been confirmed to be highly expressed in inflammation diseases and can block the activity of destructive enzymes related to inflammation 24. Abnormal expression of elafin has been reported in breast cancer, ovarian cancer 22 and melanoma 25, and is usually low expressed in these tumor tissues. Indeed, previous report indicate that overexpression of elafin may indicate chemotherapy resistance 26 but the relationship with EGFR TKIs resistance has not been reported. Our findings suggest that the downregulation of elafin may be potentially related to gefitinib resistance. S100A8 belongs to the S100 protein family, which is closely associated with the regulation of Ca^2+^ in cells, thereby involving a variety of cell functions including cell proliferation, differentiation, motility, and apoptosis 27. Research shows that the expression of S100 protein is dysregulated in almost all types of human cancer 28. Meanwhile, it has been reported that the down-regulation of S100A8 expression may indicate chemotherapy resistance 29, but whether it is involved in EGFR TKIs resistance remains unclear. Compared with the first two genes, the relationship between AXL and EGFR TKIs resistance has been widely reported. AXL is a member of the TAM family, and it transduces signal through the high-affinity ligand growth arrest-specific protein 6 (GAS6), thereby driving the proliferation, migration and invasion of tumour cells 30. It is reported that dysregulation of AXL expression is very common in the resistance of tumor cells to EGFR TKIs, the up-regulation of AXL can be detected after the first generation EGFR TKIs gefitinib, erlotinib 31 and the third generation EGFR TKI Osimertinib 32 acquired resistance. This is consistent with the results of our study. However, the role of AXL in drug resistance remains unclear, the underlying mechanism may be related to the combination of AXL and other receptor tyrosine kinases (RTKs, including EGFR, ErbB receptor family members, MET and PDFGR) to promote EGFR-induced signaling into downstream 30, 33, there are also reports that the expression changes of AXL are highly relevant to the occurrence of epithelial-mesenchymal transition (EMT) 31, 34. The current research results of combination treatment of AXL inhibitors and EGFR TKIs in NSCLC indicate that AXL is a new target for reversing drug resistance 35, 36, and AXL small molecule inhibitors are also under development and testing. PNPLA4 plays an important role in catalyzing the hydrolysis of triglycerides and the metabolism of retinol-ester in the body 37. Retinol and its related compounds may be involved in the occurrence and development of multiple malignant tumors 38, 39, and some studies have also reported that cellular lipid metabolism is related to the resistance of multiple tumor inhibitors 40, 41. However, there is no relevant report on the direct involvement of PNPLA4 in EGFR TKIs resistance, and further research is needed.

Through GO and KEGG pathway enrichment analysis of the key module in WGCNA, we found out the biological functions and possible pathways related to gefitinib resistance. Our analyses revealed that a large proportion of these co-expressed genes are mainly classified in the biological processes of protein production and transport, which are closely related to cell growth and proliferation. KEGG pathway enrichment analysis revealed that in addition to some pathways related to protein production and transport, there are also some neurodegenerative pathways (including "Parkinson's disease", "Alzheimer's disease"), which shows that gefitinib resistance and neurodegenerative diseases are potentially related in mechanism. Next, we performed GSEA to further explore potential mechanism of 4 hub genes. The results revealed that these genes play a role by influencing cell proliferation and cycle, and some metabolic-related pathways such as steroid biosynthesis and selenoamino acid metabolism have also been enriched.

Base on the above results, we further predicted the small molecule drugs that may reverse the resistance of gefitinib, and finally discovered 9 small molecule drugs. Similar to the above-described results, protein synthesis inhibitors exert essential roles in reversing gefitinib resistance. In this class of drugs, three small molecule drugs have been screened, including Emetine, cephaeline and homoharringtonine, among which the antitumor effects of Emetine have been widely reported, the mechanisms are mostly related to inducing apoptosis and blocking the cell cycle 42. Our *in vitro* verification results also show that emetine and cephaeline can significantly increase the sensitivity of drug-resistant cells to gefitinib, and the molecular mechanism may be related to the regulation of PI3 and S100A8. Besides, it is reported that PU-H71 43, anisomycin 44 and lasalocid 45 also have a tumor suppressive role, but comparatively less research directed to reversing drug resistance.

In conclusion, through several bioinformatics analyses, we have identified 4 hub genes related to gefitinib resistance in NSCLC, including PI3, S100A8, AXL and PNPLA4. They function mainly by influencing cell proliferation and cycle. Besides, we have also identified that protein synthesis inhibitors such as Emetine, cephaeline, and homoharringtonine may have potential therapeutic effects on gefitinib resistance.

## Figures and Tables

**Table 1:** Small molecule drugs identified by Connectivity Map.

Rank	Name	Description	Median Score
1	Emetine	Protein synthesis inhibitor	-86.92
2	PU-H71	HSP inhibitor	-79.05
3	cephaeline	Protein synthesis inhibitor	-75.78
4	anisomycin	DNA synthesis inhibitor	-72.85
5	HLI-373	MDM inhibitor	-69.94
6	diazepam	Benzodiazepine receptor agonist	-65.09
7	mepireserpate	Catecholamine depleting sympatholytic	-63.5
8	lasalocid	Bacterial permeability inducer	-62.58
9	homoharringtonine	Protein synthesis inhibitor	-62.47
10	cyanopindolol	Adrenergic receptor antagonist	-60.23

## Abbreviations

EGFR: epidermal growth factor receptor
EGFR TKIs: epidermal growth factor receptor tyrosine kinase inhibitors
NSCLC: non-small cell lung cancer
WGCNA: weighted gene co-expression network analysis
GO: Gene Ontology
KEGG: Kyoto Encyclopedia of Genes and Genomes
DEG: differentially expressed genes
PPI: protein-protein Interaction
GSEA: gene set enrichment analysis
TOM: topological overlap matrix
GS: gene significance
MM: module membership
